# 分子印迹聚合物：新型绿色制备方法与前沿应用综述

**DOI:** 10.3724/SP.J.1123.2025.06010

**Published:** 2026-02-08

**Authors:** Fei LI, Baolin JIA, Qiao HU, Xiwen HE, Langxing CHEN, Yukui ZHANG

**Affiliations:** 1. 山东师范大学化学化工与材料科学学院，山东 济南 250000; 1. College of Chemical Engineering and Materials Science，Shandong Normal University，Jinan 250000，China; 2. 南开大学化学学院，生物传感与分子识别 天津市重点实验室，天津 300071; 2. College of Chemistry，Tianjin Key Laboratory of Biosensing and Molecular Recognition，Tianjin 300071，China; 3. 中国科学院大连化学物理研究所，辽宁 大连 116023; 3. Dalian Institute of Chemical Physics，Chinese Academy of Science，Dalian 116023，China

**Keywords:** 分子印迹聚合物, 绿色化学, 环境监测, 食品安全, 生物医学, 综述, molecularly imprinted polymers, green chemistry, environmental monitoring, food safety, biomedicine, review

## Abstract

分子印迹技术是一种新兴的，通过模拟抗体-抗原或酶-底物之间的相互作用，制备对模板分子具有特异性识别功能的分子印迹聚合物（MIPs）的技术。传统MIPs的制备方法因受限于形状不均匀、分子识别构象选择少、聚合随机不可控、威胁环境安全等弊端而面临严峻挑战，合成方法亟待革新。近年来，随着绿色化学理念的提出与绿色合成方法的发展，分子印迹聚合物逐渐向更绿色的方向迁移。绿色分子印迹聚合物（GMIPs）的制备旨在替代传统方法，减少在合成过程中溶剂的使用和废液的产生、使用安全无毒的试剂和溶剂、发展高效合成方法提高能源效率等。采用的绿色溶剂水、超临界二氧化碳、低共熔溶剂和离子液体替代传统MIPs合成中使用的有机溶剂；具有生物相容性、环境友好型的功能单体壳聚糖、纤维素、衣康酸、多巴胺和环糊精在MIPs的制备中获得更多的应用。另外，MIPs的制备技术逐步向资源节约和环境友好型过渡，绿色沉淀聚合法、微波辅助合成、超临界流体技术、超声辅助聚合以及计算机模拟辅助设计等新型合成方法的出现，实现了MIPs制备方法绿色化的快速推进。这些新型制备方法通过精准调控反应条件、降低能耗、减少有害副产物，显著提高了MIPs的功能性和环境兼容性，不仅优化了MIPs的合成效率，还为解决传统方法在形态控制和规模化生产中的瓶颈问题提供了新思路。绿色分子印迹聚合物凭借其高选择性、稳定性和可定制性，在多个前沿领域展现出突破性应用。本文综述了近年来绿色分子印迹聚合物的新型制备方法及其在环境监测、食品安全、生物医学等领域的应用情况，并对绿色分子印迹聚合物的发展进行了展望。

## Abstract (en)

Molecular imprinting technology is an emerging technique that achieves specific recognition of imprinted molecules by simulating the interactions between antibody and antigen or between enzyme and substrate. The core of this technology lies in the preparation of molecularly imprinted polymers （MIPs）. However， traditional preparation methods of MIPs face severe challenges due to drawbacks such as uneven morphology， limited conformational choices for molecular recognition， random and uncontrollable polymerization， and environmental safety hazards， making the innovation of synthesis methods urgent. In recent years， with the proposal of green chemistry concepts and the development of green synthesis methods， the preparation technology of MIPs has gradually transitioned towards resource-saving and environment-friendly directions. The preparation of green molecularly imprinted polymers （GMIPs） aims to replace traditional methods by reducing the use of solvents and the generation of waste liquids during the synthesis process， employing safe and non-toxic reagents and solvents， and developing efficient synthesis methods to improve energy efficiency. Green solvents such as water， supercritical carbon dioxide， deep eutectic solvents， and ionic liquids are used to replace organic solvents in the synthesis of traditional MIPs. Functional monomers with biocompatibility and environmental friendliness， including chitosan， cellulose， itaconic acid， dopamine， and cyclodextrin， have found increasing applications in the preparation of MIPs. In addition， the preparation technology of MIPs is gradually transitioning towards resource conservation and environmental friendliness. The development of novel synthesis methods such as green precipitation polymerization， microwave-assisted synthesis， supercritical fluid technology， ultrasound-assisted polymerization， and computer simulation-assisted design and characterization has promoted the popularity of GMIPs preparation methods. These novel preparation methods significantly improve the functionality and environmental compatibility of MIPs by precisely regulating reaction conditions， reducing energy consumption， and minimizing harmful by-products. They not only optimize the synthesis efficiency of MIPs， but also provide new ideas for solving the bottlenecks of traditional methods in morphology control and large-scale production. GMIPs， with their high selectivity， stability， and tunability， have shown breakthrough applications in multiple frontier fields. For example， in environmental monitoring， GMIPs are applied to detect heavy metal ions （such as lead and arsenic）， organic pollutants （such as pesticides and antibiotics）， and explosives in aqueous environments. In the field of food safety analysis， GMIPs enable efficient enrichment and detection of trace pollutants （such as pesticide residues， veterinary drugs， and mycotoxins） in food matrices， significantly outperforming traditional methods. In biomedical applications， GMIPs are developed as drug controlled-release systems， biomarker detection platforms， and targeted therapeutic carriers. In addition， the efficient performance of GMIPs in sample pretreatment （such as solid-phase extraction） further reduces analysis costs and reduces reliance on organic solvents. This paper reviews the novel preparation methods of GMIPs and their applications in environmental monitoring， food safety， and biomedicine in recent years， and provides an outlook on the development of GMIPs.

分子印迹技术（MIT）是一种新兴的分子识别技术^［1］^，是模拟酶-底物或抗体-抗原之间的相互作用，对印迹分子（也称模板分子）进行专一识别的技术，具有特异性识别“钥匙（模板）”的能力^［2］^，而该技术的核心又在于分子印迹聚合物（MIPs）的制备。而传统MIPs的制备方案存在形状不均匀、分子识别的构象选择少、聚合随机不可控^［3］^等诸多弊端，尤其是其对环境安全颇具威胁性，这也决定了传统合成方法必然面临转型升级。

近年来，MIPs的合成技术开始逐渐向绿色化学^［4，5］^方向转移。绿色化学可以理解为环境友好型化学，即在合成过程中尽可能减少环境污染的风险。绿色分析化学（GAC）提出了12条相应原则，其中包括最小化能源使用、优先使用可再生来源的试剂等^［6］^；又如，在样品制备时的预防废物产生、使用安全无毒的试剂和溶剂、提高能源效率、减少衍生化步骤^［7］^。此外还有14项绿色MIT原则的构想，包括使用可再生和无害的合成材料的前驱体、选择合适的聚合方法、提高印迹效率等^［8］^。同时，为了实现有效监测，分析生态尺度（analytical eco-scale）、绿色分析程序指数（GAPI）和分析绿色度指标（AGREE）等检测指标^［9］^也应运而生。超声辅助法、微波辅助法、表面印迹技术^［10-13］^等新型绿色合成方法层出不穷并且逐渐趋于成熟。这些理论与实践的结合使MIT突破了末端治理的局限，在碳中和背景下，构建了更加绿色的MIPs制备范式。

在应用方面，绿色分子印迹聚合物（GMIPs）正在逐步突破传统检测与分离的边界，向动态传感和生物工程深度拓展。化学和生物传感、颜色识别测试系统、磁性生物分离、药物输送、酶固定化、刺激响应技术^［14-17］^等前沿领域都出现了MIPs的身影，例如，MIPs具有特定的识别位点，而荧光碳点（CDs）具有优异的光学特性、良好的水溶性和生物相容性，将MIPs与CDs结合，有效提高了荧光传感器的选择性、灵敏度和抗干扰能力^［18］^。GMIPs渐渐从静态识别材料进化为具有环境交互能力的智能系统，正在重塑精准医学与碳中和技术的底层逻辑。

本文综述了近年来MIPs的绿色沉淀聚合法、微波辅助制备、超临界流体技术等新型绿色制备方法以及其在环境监测、食品安全和生物医学等前沿领域的具体应用。

## 1 绿色分子印迹聚合物的制备方法

### 1.1 分子印迹聚合物制备原理

MIPs是一种对模板分子具有高度选择性的聚合物，MIPs的制备过程中包含模板、交联剂、功能单体、引发剂和溶剂^［19］^等化学物质。因此，MIPs的性能主要取决于配方的类型和成分量。具体合成步骤如下：（1）通过模板分子和功能单体的预排列或自组装形成预聚合配合物；（2）加入交联剂和引发剂，然后通过光或热聚合，固定步骤1中形成的配合物的结构；（3）选择合适的溶剂去除模板分子，获得MIPs^［20］^（图1）。为了进行比较分析，以相同的方式制备非印迹聚合物（NIPs），制备过程中没有模板分子，通常NIPs作为参考聚合物。

**图1:**
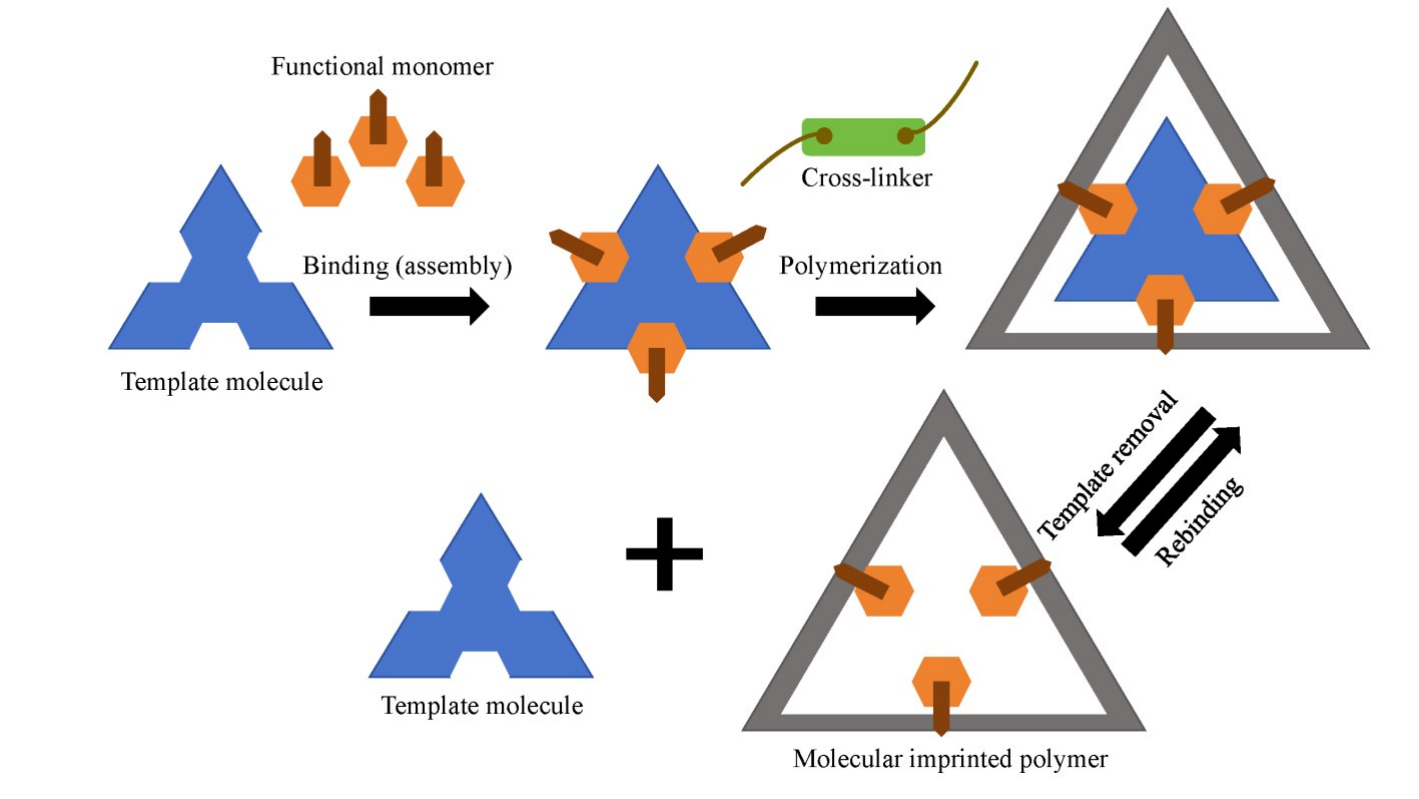
分子印迹聚合物制备的基本步骤

### 1.2 分子印迹制备中的绿色试剂

绿色分子印迹聚合物的制备旨在替代传统方法，减少在合成过程中污染物的产生、使用安全无毒的试剂和溶剂、提高能源效率等。对传统MIPs合成中使用的溶剂、模板、功能性单体和洗脱溶剂使用环境友好型替代品。

#### 1.2.1 功能单体

甲基丙烯酸、丙烯酰胺、乙烯基咪唑和乙烯基吡啶是传统MIPs合成中广泛应用的功能单体，它们能够与多种模板分子产生相互作用^［17］^。然而，许多这类经典单体具有高毒性，且对某些分析物的选择性可能会降低。从绿色分析的角度来看，一些生物聚合物在MIPs合成中作为单体表现出优异的特性。壳聚糖、纤维素、衣康酸、多巴胺和环糊精因具有无毒、亲水性、生物相容性、可降解性是经典单体的一些替代选择。它们常含有胺基、羟基等官能团，这些官能团还允许对这些生物聚合物进行化学修饰，旨在提高其吸附能力，同时仍能与更多样化的模板分子产生相互作用^［21，22］^。

壳聚糖是从甲壳类动物外壳中提取的天然多糖，具有可再生、生物可降解等特性，它本身含丰富氨基和羟基官能团^［23］^。作为生物聚合物，壳聚糖的官能团具有极强的吸附重金属、染料、药物甚至蛋白质的能力。此外，这些官能团还可用于壳聚糖的化学衍生化修饰，有助于增强其吸附能力及MIPs合成过程中所用试剂之间的相互作用。因此，壳聚糖是一种可生物降解且具有生物相容性的分子，可在无有机溶剂的条件下实现低危害的MIP合成^［24，25］^。基于壳聚糖的MIPs已被应用于控制核黄素释放的药物释放系统^［26］^，以及用于去除水中镉离子的离子印迹聚合物^［27］^。

纤维素是自然界中最丰富的生物聚合物，它由D-葡萄糖通过*β*1-4糖苷键连接形成线性链，作为功能单体已被用于MIPs的制备中^［22］^。纤维素分子在极端pH条件下表现出高稳定性，且在水中可生物降解，其中羧甲基纤维素因其高吸附容量、低成本和低环境影响等特性，已被应用于合成含硫印迹聚合物，并从水溶液中去除汞^［28］^。

衣康酸可通过微生物发酵法（如黑曲霉）从可再生生物质原料中规模化生产，符合绿色化学的原料可持续性原则，它含双羧基结构，能与氨基或羟基模板（如抗生素、多巴胺）形成强氢键^［29］^，替代石油基丙烯酸，聚合后残留在水相中可被微生物分解，生态毒性趋近于0。

自从Lee等^［30］^用多巴胺分子模仿贻贝黏附蛋白的成分，在弱碱性水溶液（pH 8.5）中，能稳定地固定在各种基质上（惰性金属、金属氧化物、聚合物、半导体和陶瓷等）自聚合形成聚多巴胺（PDA）。聚多巴胺作为新型仿生材料制备过程简单，绿色环保，生物相容性好，适用于无机、有机、金属等各种类型的基质。另外，聚多巴胺为材料的表面功能化提供了多种高效的修饰方法，聚多巴胺可以提供下一步反应的位点。多巴胺作为功能单体和交联剂已应用于有机小分子和生物大分子的分子印迹聚合物的制备^［31-35］^。与传统分子印迹聚合物相比，聚多巴胺分子印迹聚合物具有高亲水性，以及优秀的生物相容性，对支撑基质有强的吸附力，分子印迹层含有数量众多的功能基团，合成方法简单，而且聚多巴胺分子印迹层厚度可控。相对于有机小分子模板，聚多巴胺功能单体更适合生物大分子蛋白质体系。

另一种更绿色的单体替代品是环糊精，其来源于马铃薯和玉米的淀粉，被认为是绿色可再生物质。其中*β*-环糊精是由*α*-D-吡喃葡萄糖单元环化形成的生物聚合物，其官能团可被修饰以提高选择性。*β*-环糊精聚合物形成具有典型疏水空腔的亲水外表面，这增强了环糊精聚合物的双重识别能力^［36］^。基于环糊精具有独特的功能特性，将环糊精的特性与分子印迹聚合物的设计和合成相结合，为分子印迹聚合物带来了诸多优势，包括增强目标分析物的吸附能力、稳定性、选择性和可重复使用性，以及在水介质中发挥作用的能力。此外，在分子印迹聚合物的合成中使用环糊精，能够最大限度地降低传统分子印迹聚合物所带来的潜在危害。尽管目前环糊精分子印迹聚合物存在一些局限性，例如结合亲和力较低以及会受到基质成分的干扰，但相关研究正专注于开发新的设计和技术来解决这些问题^［37，38］^。

#### 1.2.2 溶剂

在MIPs制备中，模板分子、功能单体和交联剂需要分散在溶剂中，同时溶剂作为致孔试剂进而影响孔的形成，对MIPs的形貌和结构有显著影响^［39］^。若致孔溶剂用量过大，可能导致聚合物的空间结构和机械性能变差，从而影响其吸附性能。而致孔溶剂用量较低时，会使交联度更高，形成的印迹空穴更致密^［40］^。此外，模板与致孔溶剂之间的相互作用在很大程度上受极性影响。低介电常数的溶剂倾向于稳定模板与单体之间的氢键或静电相互作用。因此，甲苯、氯仿、乙腈和二氯甲烷是最常用的溶剂，然而，这些溶剂具有较大毒性，因此需要使用更环保的替代溶剂。就溶剂而言，常采用的绿色溶剂有水、超临界二氧化碳、室温离子液体和低共熔溶剂等^［22］^。水是最环保的溶剂，无毒性，成本几乎可以忽略，用水替代传统有机溶剂，降低了挥发性有机化合物（VOC）的排放，清洗中多次使用水去除模板分子和杂质，也减少了有害溶剂的使用，甚至能使部分材料具有更好的生物相容性^［41-43］^。例如Li等^［43］^提出了一种温和条件下于水相中合成环磷酸腺苷磁性印迹聚合物的绿色策略。该法采用水为制备溶剂，生物相容性良好的壳聚糖为功能单体和交联剂，制备过程简单，毒性低，价格便宜。制备的绿色磁性印迹纳米材料成功应用于实际中药样品冬枣中环磷酸腺苷的吸附提取。

超临界二氧化碳^［44］^无毒、不可燃、价格低廉且易于回收，作为反应介质又作为致孔剂，有效稳定模板-单体复合物，同时通过超临界状态的高扩散性可以实现模板分子的高效脱附，避免了有机溶剂的残留，产物直接获得无溶剂的自由流动粉末。

近年来，基于室温离子液体（RTILs）和低共熔溶剂（DESs）的分子印迹聚合物已成为分子印迹技术领域中一个活跃且令人关注的研究方向^［20，22，45-47］^。RTILs和DESs可用作溶剂、功能单体、致孔剂、孔模板等在分子印迹聚合物的制备过程中发挥着多种不同的作用。基于RTILs和DESs的MIPs克服了传统MIPs的诸多缺点。例如，RTILs和DESs有助于提升大分子MIPs的效果。使用RTILs作为致孔剂可减少MIPs的收缩或溶胀现象。此外，事实证明，基于RTILs和DESs的MIPs在水介质中能保持优异的性能。与传统MIPs相比，这些新型材料具有更出色的特异性识别能力。然而，室温离子液体的价格十分昂贵。这些缺点在一定程度上限制了其应用。为了克服室温离子液体的不足，DESs作为新一代绿色溶剂应运而生，DESs不仅具备室温离子液体的特性，而且更加廉价、安全。针对基于DESs的MIPs这类新型材料的研究相对较少。另外，RTILs和DESs在MIPs合成过程中的作用机制尚未得到深入研究。寻找能够替代传统有机溶剂的绿色试剂已成为分子印迹技术的研究趋势。

### 1.3 分子印迹聚合物的绿色制备方法

#### 1.3.1 绿色沉淀聚合法

绿色沉淀聚合法（GPP）通过在溶液中引发单体的聚合反应来形成聚合物颗粒（图2）^［48，49］^。与传统方法依赖甲苯、乙腈等有机溶剂不同，该技术通过水溶性单体与纯水或低浓度有机溶剂体系的协同作用实现聚合反应，使溶剂用量大大降低；水相体系有效抑制挥发性有机物的排放，且反应条件较传统热引发聚合降低能耗30%~50%，同时避免了有机废液处理难题，符合原子经济性原则。在沉淀聚合过程中，单体和引发剂在溶剂中混合，随后引发聚合反应。随着聚合反应的进行，生成的聚合物分子链逐渐增长，当聚合物分子链的长度达到一定程度时，它们会从溶液中沉淀出来形成固体颗粒，这种颠覆性溶剂体系与动态交联机制实现了环境友好性与功能性的双重突破。例如，MIP（MAA）是通过使用甲基丙烯酸（MAA）作为功能单体合成的，而MIP（AAm）和MIP（2-VP）分别使用了丙烯酰胺（AAm）和2-乙烯基吡啶（2-VP）作为功能单体^［50］^。

**图2:**
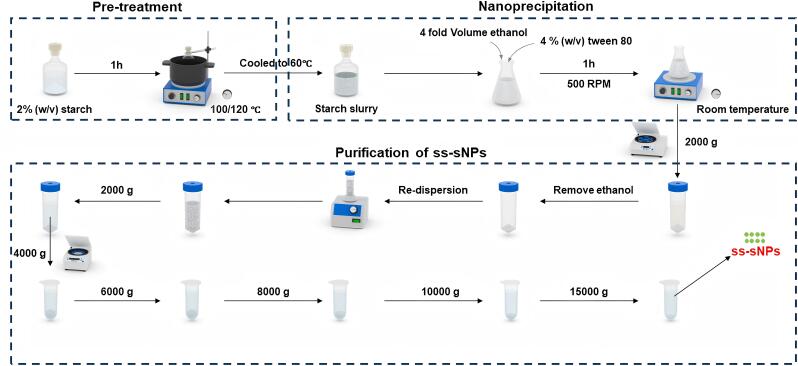
制备小粒径淀粉纳米颗粒的示意图^［48］^

肌酐是肌酸代谢的终产物，常作为临床指标用于评估肾脏和肌肉功能^［51，52］^，肌酸在水中可转化为肌酐，且其浓度随红细胞年龄增长而降低，因此肌酸和肌酐的分离对于准确检测肌酐水平至关重要。Min等^［53］^将肌酐与丙烯酸混合形成复合物，然后加入丙烯酰胺和交联剂乙二醇二甲基丙烯酸酯（EGDMA），通过聚合反应成功制备MIPs，在紫外分光光度计测量吸附量，计算印迹因子（IF）和表观选择性等操作后验证制得了对肌酐的表观选择性显著高于其类似物肌酸、胞嘧啶和3-氨基-1，2，4-三唑的分子印迹聚合物。

Wang等^［54］^使用了聚（9-乙烯咔唑）（PVK）和聚（苯乙烯-顺丁烯二酸酐）（PSMA）两种聚合物，以及对扑热息痛（PCM）、尿酸（UA）、L-苯丙氨酸（PA）和腺嘌呤（A）等化学物质，通过共沉淀方法成功制备了对扑热息痛具有特异性识别能力的分子印迹荧光聚合物纳米颗粒PVK/PSMA-PCM NPs。

Zeng等^［55］^采用沉淀聚合方法，以MAA为单体，EGDMA为交联剂，制备了水兼容的微米级MIPs，又通过比较模板与单体物质的量比为1∶5、1∶10和1∶15时的3种情况，发现1∶10的比例下MIP具有最高的吸附容量和印迹因子，优化了泰乐菌素的高效纯化方案，解决了传统的溶剂萃取法无法满足环保要求的弊病。

巴氯芬（baclofen）是一种口服合成镇静剂^［56］^，用于治疗肌肉痉挛和多种神经系统疾病，由于其在生物体内的快速吸收和代谢，以及在尿液中的高排泄率，准确测定生物样本中的巴氯芬含量对于临床治疗和药物监测具有重要意义。Abbas团队^［57］^首先合成了Fe_3_O_4_@SiO_2_磁性纳米颗粒，然后在其表面修饰了壳聚糖层，制备出超顺磁性分子印迹生物聚合物（SMIBP），再通过添加NaOH溶液，使壳聚糖沉淀在Fe_3_O_4_@SiO_2_表面，形成一种水兼容的SMIBP。吸附试验结果显示SMIBP具有良好的磁性特征，能够通过外部磁场方便快捷地收集。

#### 1.3.2 微波辅助制备

微波辅助制备分子印迹聚合物（MA-MIPs）通过电磁场与分子相互作用的精准调控（图3）^［58，59］^，开创了高效节能的绿色合成新维度。微波辐射可以提供比传统加热方法更快的反应时间，并且能够直接吸收微波能量，从而实现更快速的加热，将反应时间缩短至1~4 h，显著减少碳足迹，契合绿色化学的污染预防（pollution prevention）及原子经济性（atom economy）原则。此外，微波热辐射通常比传统加热方法更节能，因为它直接作用于反应物，减少了热量损失到周围环境的风险，从而实现了均匀加热，减少了热点的风险，提高了聚合物产品的质量。并且微波合成一般是在水性介质中进行的，避免了有机溶剂的使用，从而显著提高了聚合物的产率^［60］^。微波辅助制备方法已应用于金属离子、抗生素、环境污染物的MIPs的制备^［61-69］^。表1为微波辅助制备MIPs的应用示例。

**图3:**
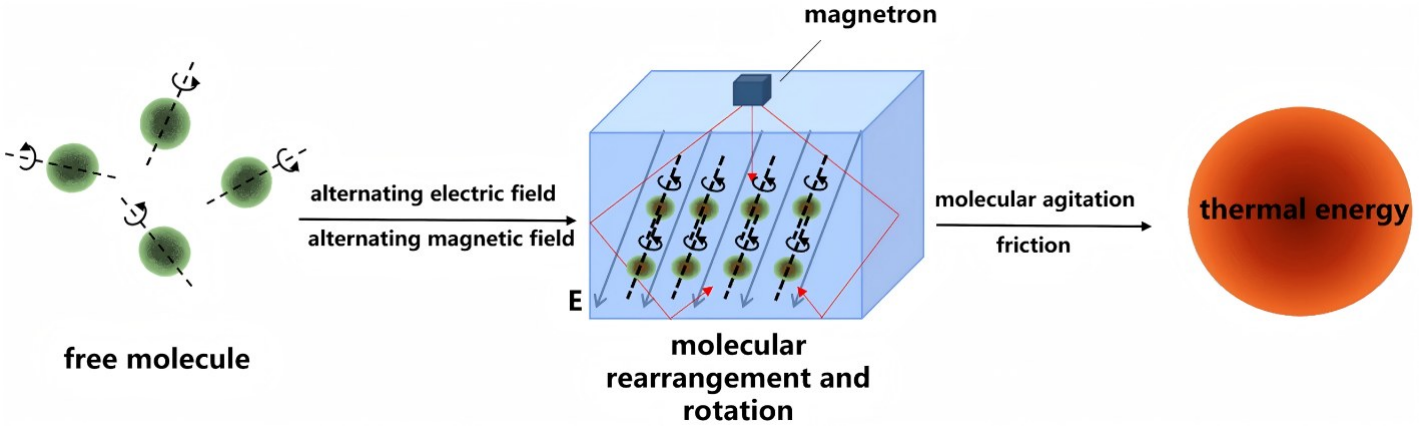
微波技术的原理^［59］^

**表1:** 微波辅助制备MIPs的应用示例

Sample	Analytes	Main material characterization	Reference
Water	Pb（Ⅱ）， As（Ⅴ）	FT-IR， XRD， BET	［^61^］
Water	sulfamethoxazole	HRSTEM，FT-IR， TGA	［^62^］
Urine	perphenazine	HRTEM， FESEM， UV/vis	［^63^］
Water	4-nitrophenol	SEM， FT-IR， TEM	［^64^］
Water	nitrophenol	SEM， BET， TG	［^65^］
Milk	tetracycline	TEM， SEM， FS	［^66^］
Milk	florfenicol	SEM， FT-IR	［^67^］
Water	malachite green	FT-IR， SEM， EDS	［^68^］
Water	Cd（Ⅱ）， As（V）	FT-IR， XRD， TG	［^69^］

FT-IR： Fourier transform infrared spectroscopy； XRD： X-ray diffraction； BET： Brunauer-Emmett-Teller； HRSTEM： high resolution scanning transmission electron microscopy； TGA： thermogravimetric analysis； HRTEM： high resolution transmission electron microscopy； FESEM： field emission scanning electron microscope； UV/vis： ultraviolet-visible spectroscopy； TEM： transmission electron microscope； TG： thermogravimetry； FS： fluorescent sensing； EDS： energy dispersive X-ray spectroscopy.

微波加热因其精确的温度和压力控制，在聚合反应中得到了广泛应用。微波合成器通常为单模微波系统，提供了精确的温度和压力控制，从而提高了反应的安全性和可重复性^［70］^。尽管其目前在放大反应和工业应用方面仍存在挑战，但随着研究的深入和技术的进步，微波辅助合成将在分子印迹聚合物制备中成为不可或缺的工具。

#### 1.3.3 超临界流体技术

超临界流体技术（supercritical fluid technology， SFT）是一种利用物质在超临界状态（即温度和压力超过其临界点）下的独特性质进行科学研究和工业应用的技术^［71，72］^。超临界流体既不是传统的气体，也不是液体，而是兼具两者的特性。超临界流体，如超临界二氧化碳（ScCO_2_），作为惰性、可循环的反应介质，可完全消除有机溶剂使用、洗脱等高污染后处理工序，使体系符合绿色MIT原则，其通过颠覆性介质工程与热力学精准调控，重新定义了MIPs的绿色制备边界。超临界流体技术在分子印迹聚合物制备方面的应用颇具创新性，例如，ScCO₂作为溶剂和孔隙剂时，利用超临界流体高扩散性、低黏度等特性来提高模板从聚合物基质中的提取效率，并且可以在没有溶剂的情况下获得具有控制形态和孔隙率的MIPs粉末^［73］^。

Hun团队^［74］^使用甲基丙烯酸甲酯（MMA）作为第三单体，MAA作为功能单体，双酚A（BPA）、2，4-二氯苯氧乙酸（2，4-D）作为模板，EGDMA作为交联剂，利用超临界流体技术合成了BPA和2，4-D的高选择性分子印迹聚合物，避免使用有害有机溶剂。多种评估方法都确认了MIPs的吸附性能优于目标分子和其他材料，这表明超临界聚合过程可以作为一种制备高功能聚合物的新方法。Marcelo等^［75］^使用了EGDMA作为交联剂，衣康酸（IA）作为功能单体，甲硝唑（MZ）作为模板分子，偶氮二异丁腈（AIBN）作为引发剂，合成了具有pH响应性的MIPs。随后模拟口服给药情况下的性能表明，MIPs在模拟口服给药情况下对甲硝唑（MZ）具有更高的亲和力和更优异的药物装载能力。

#### 1.3.4 超声辅助制备

超声辅助制备分子印迹聚合物（UA-MIPs）是一种利用超声能量来加速化学反应的方法^［76-79］^。具体来说，超声波的使用导致了小气泡的形成和崩溃，这增加了介质中物种的溶解度、扩散性、穿透性和传输性。与传统聚合过程相比，超声辅助聚合具有更快的反应速率、更均匀的链增长、更高的产率和更温和的条件等优势^［80］^，超声辅助制备分子印迹聚合物的绿色性主要体现在其显著降低环境负荷的工艺革新：超声技术通过产生微射流和冲击波加速分子扩散与传质，使预组装体系中功能单体与模板分子的定向结合效率提升30%~50%，从而减少50%以上的交联剂与引发剂用量，可将反应时间缩短至传统方法的1/3~1/2，同步降低溶剂挥发与能源消耗；此外，超声引发的局部高温高压环境可替代部分化学活化步骤，使水相或低毒溶剂体系的应用成为可能。这种制备方法通过声化学效应与界面工程的深度融合，开创了分子识别材料的高效精准制造新模式。

黄曲霉毒素（AF）是一类由多种曲霉菌产生的次级代谢产物^［81］^，对人类和动物健康构成严重威胁，由于AF在固体材料中的提取通常需要使用特定的提取方法和溶剂，例如，在高效液相色谱法（HPLC）检测AF时，通常会使用有机溶剂如甲醇、乙腈或它们的混合物进行提取，因此迫切需要开发一种更加高效、选择性更强的方法。Jayasinghe等^［82］^在研究中使用超声辅助提取技术，以体积比60∶40的乙腈/0.1 mol/L KH_2_PO_4_溶液作为提取溶剂，通过40%的振幅连续超声7 min，实现了从鱼饲料中高效提取AF，并通过MIP-μ-SPE实现高效率的预浓缩。与此类似的是Niranjan团队^［83］^在MMIPs/MNIPs的制备时，使用超声波辅助进行聚合反应，经过后续处理，成功合成了一种对氯吡硫磷具有高选择性和吸附能力且具有良好的重复使用性的磁性分子印迹聚合物（MMIPs）。

*β*-阻滞剂索他洛尔（SOT）是治疗心血管疾病的常用药物，其在生物流体中的测定对于开展药物相互作用研究及临床药物监测均具有重要意义^［84］^。Ansari等^［85］^采用超声辅助分散固相微萃取技术，结合高效液相色谱-紫外检测方法，对生物流体样本中的SOT进行选择性提取和测定，制备出的分子印迹聚合物对生物流体样本中SOT的选择性和有效预浓缩表现出色，在吸附容量和磁性方面也优于传统球形分子印迹聚合物。与此类似的，Ansari等^［86］^报道了使用了包括卡培他滨（CAP）、吉西他滨、多西他赛等在内的多种化学试剂和材料，通过实验设计优化了提取条件，如确定最佳超声时间，获得最高的响应，该方法结合了高效的超声辅助分散固相微萃取技术与简单的分析测定，为不同基质中各种化合物的分析提供了一种快速、可靠、成本效益高的方法。

#### 1.3.5 计算机辅助制备

计算机模拟辅助方法是一种利用计算机技术作为载体，结合量子力学和统计力学的理论基础作为工具的交叉学科方法^［87，88］^。该方法通过计算和比较分子间相互作用的形式与能量关系，模拟分子的静态结构和动态运动变化，以有效解释分子水平上的作用机制。传统方法依赖试错法筛选功能单体、模板分子与交联剂配比，需进行数十次重复合成与表征，产生大量有机溶剂（如氯仿、二甲基亚砜）及未反应单体的废弃污染。而计算机辅助技术可预先预测模板-单体复合物的结合能、空间匹配度及聚合反应路径，将功能单体筛选效率提升5~8倍，使溶剂使用量减少40%~60%，同时避免有毒试剂的盲目引入。此外，基于响应面分析或人工神经网络的工艺优化模型，能精确预测最佳引发剂浓度、聚合温度及时间，将能耗降低30%~50%，并通过虚拟模板技术减少生物毒性模板分子的实际使用，这种“预测-验证”循环模式深度融合绿色化学的原子经济性与预防性原则，这种方法操作简单，不受空间环境限制，计算准确且高效，能够显著降低MIPs聚合条件优化的成本（图4）^［89-92］^。表2列出的一些计算机辅助制备MIPs在环境监测、食品安全领域的应用示例。

**图4:**
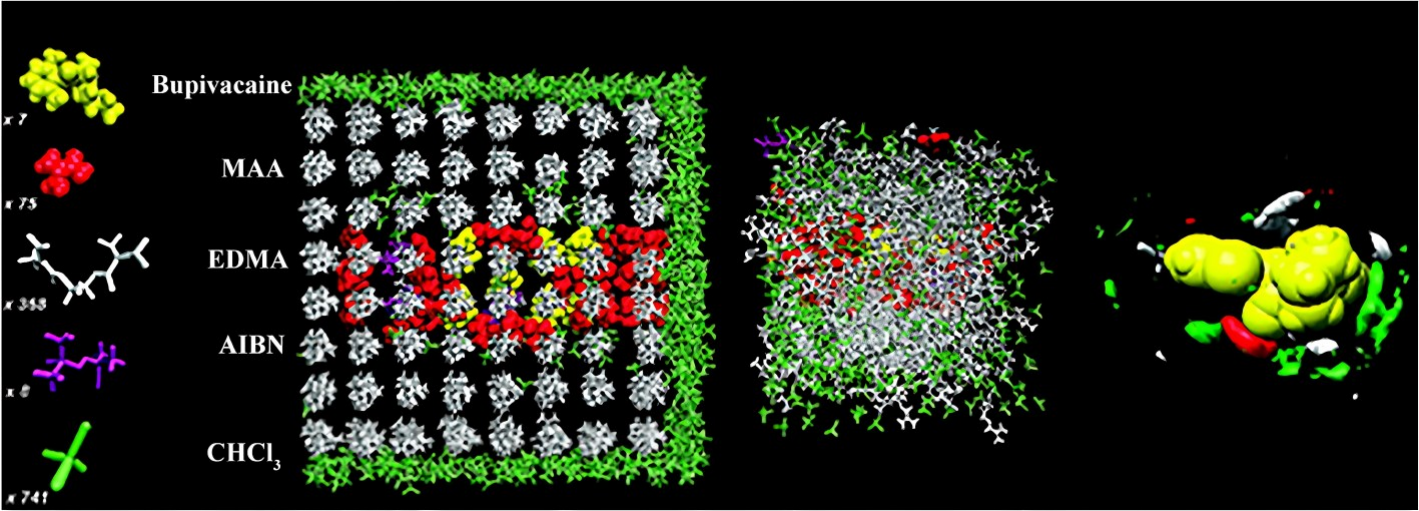
分子动力学计算模拟分子印迹预组装示意图^［89］^

**表2:** 计算机辅助制备MIPs的应用示例

Analyte	Main raw materials	Application prospect	Ref.
Bilobalide	MAM， TMPTA， ACN	biomedicine	［^93^］
New recreational drugs	EGCG， ACE， NI， IA	criminal investigations	［^94^］
As（Ⅲ）	UiO-66 MOF， MAA， EGDMA	environmental monitoring	［^95^］
Triazole fungicides	MYC， TFMAA， EGDMA	food safety	［^96^］
Tolfenpyrad	FeCl_2_， VTMS， EGDMA， 2-VP， DMF	food safety	［^97^］
Thiamethoxam	AA， MAA， APV， EGDMA， AIBN	environmental monitoring	［^98^］
Hydroxycamptothecin	TEOS， EGDMA， AIBN， MeOH， CHCl₃	biomedicine	［^99^］
Estrone	IA， PEG3A， AIBN	environmental monitoring	［^100^］

MAM： methyl acrylamide； TMPTA： trihydroxymethylpropane triacrylate； ACN： acetonitrile； EGCG： catechin； ACE： acetaminophen； NI： nicotine； IA： itaconic acid； MAA： methacrylic acid； EGDMA： dimethyl methacrylate； MYC： trizole fungicides； TFMAA： 2-（trifluoromethyl）acrylic acid； VTMS： triethoxysilane； 2-VP： 2-vinylpyridine； DMF： *N，N*-dimethylformamide； AA： acroleic acid； APV： on vinyl benzoic acid； AIBN： 2，2-azo-diisobutyronitrile； PEG3A： polyethylene glycol triacrylate.

Silva等^［101］^通过分子动力学模拟和实验验证相结合的方法，成功地确定了丙烯酰胺作为东莨菪碱^［102，103］^分子印迹聚合物合成的最佳功能单体，模拟结果与实验结果高度一致。计算机辅助制备优化了传统分子印迹聚合物的制备方法，克服了合成过程中耗时且需要大量合成的缺点。借助计算工具，可缩短操作时间，并最大程度减少化学品浪费。同时准确预测最佳合成条件，是极具发展前景的合成方式。

#### 1.3.6 溶胶-凝胶法

溶胶-凝胶法（Sol-gel）是一种通过水解和缩聚反应将前驱体（如硅烷单体）转化为溶胶，再通过凝胶化形成三维网络结构的材料制备方法^［104］^，常用于制备分子印迹聚合物，具有物理刚性、化学惰性、热稳定性和亲水性能的特点。在表面活性剂介导下，可在水相环境中实现单体的均匀分散，避免有机溶剂对蛋白质结构的破坏^［105］^。具体步骤包括：单体水解→溶胶形成→凝胶化（通过pH调节或催化剂触发聚合）→模板去除（如洗涤），最终形成具有特异性识别位点的聚合物。

Sol-gel具有环境友好性、结构可控性等优势，在水相中操作，避免了使用有毒有机溶剂（如甲醇、甲苯），通过表面活性剂调控单体分散状态，形成均相印迹层，适用于复杂生物分子（如人血清白蛋白）的印迹。合成温度偏低（约50 ℃），且高重复使用性（30次左右）降低了资源消耗，高选择性减少了无效吸附导致的废液处理需求^［105，106］^。

已有研究表明，分子印迹聚合物因其形状记忆特性被广泛用于分析化学领域，作为预浓缩相可有效识别目标化合物。然而传统MIP通常使用有机溶剂合成，可能带来环境和健康风险。相比之下，Sol-gel采用水/乙醇混合溶剂体系，更符合绿色化学原则。

Chen课题组^［107，108］^以磁性Fe_3_O_4_纳米颗粒和碳纳米管为载体，先合成雌酮-硅烷复合物，采用半共价键法反应制备了雌酮MIPs。制备方法合成过程简单，且不同批次间所得物重现性好。将磁性MIPs和碳纳米管MIPs用于环境和自来水样中雌酮的去除，去除效果理想，分离过程简单快速。进一步，结合Sol-gel和可控自由基聚合反应合成了磺胺二甲嘧啶核壳结构的印迹聚合物磁性纳米球，成功用于家禽饲料中磺胺抗生素的选择性富集和分析检测，对低于国标检出限含量的样品实现有效的富集和检测，方法准确性高^［109］^。

马兜铃酸I（AAI）是一种存在于多种中药材中的化合物，尽管具有抗炎、抗疟疾和降血糖等药理活性，但其与肝癌的关联已引起广泛关注，因此，开发一种高灵敏度和高选择性的方法来检测和去除中药中的AAI成为亟待解决的问题。Li等^［110］^采用Sol-gel，以马兜铃酸为模板分子，苯基三甲氧基硅烷为功能单体，正硅酸乙酯为交联剂，乙醇/水为反应溶剂，磁性碳纳米管为载体，制备了具有均匀核-壳结构的AAI磁性分子印迹聚合物。结果表明，该绿色磁性印迹纳米材料具有一系列优点，如吸附平衡时间短（15 min），吸附能力强（18.54 µg/mg），IF=4.84。

洛克沙胂（ROX）作为一种有机砷饲料添加剂，被广泛应用于畜牧业和家禽养殖业，但其在环境中降解为高毒性无机砷物种的特性对环境和人类健康构成威胁。Li等^［111］^使用了包括四水硫酸锰、氨水、九水硫化钠、硝基酚、三乙氧基硅烷和2-甲氧基-5-硝基苯酚在内的试剂，并通过Sol-gel成功开发了一种基于分子印迹聚合物功能化的锰掺杂硫化锌量子点荧光传感器，检出限为4.34 nmol/L，线性范围为3.75×10^‒8^~6.25×10^‒7^ mol/L，竞争实验表明，其对ROX的选择性远高于其结构类似物。Manal及其团队成员^［112］^开发了一种基于分子印迹溶胶-凝胶聚合物（MIS）的廉价分析方法，用于检测白葡萄酒中的杀菌剂异丙定，并通过实验设计优化了MIS的合成条件，实现了高选择性和低检出限。

Wang等^［113］^成功开发了一种室温下的Sol-gel并将其应用于制备分子印迹电化学传感器，扫描电子显微镜、傅里叶变换红外光谱等表征手段证明该传感器具有优异的性能，可应用于动物血液中肾上腺素的选择性吸附和高灵敏检测（图5）。

**图5:**
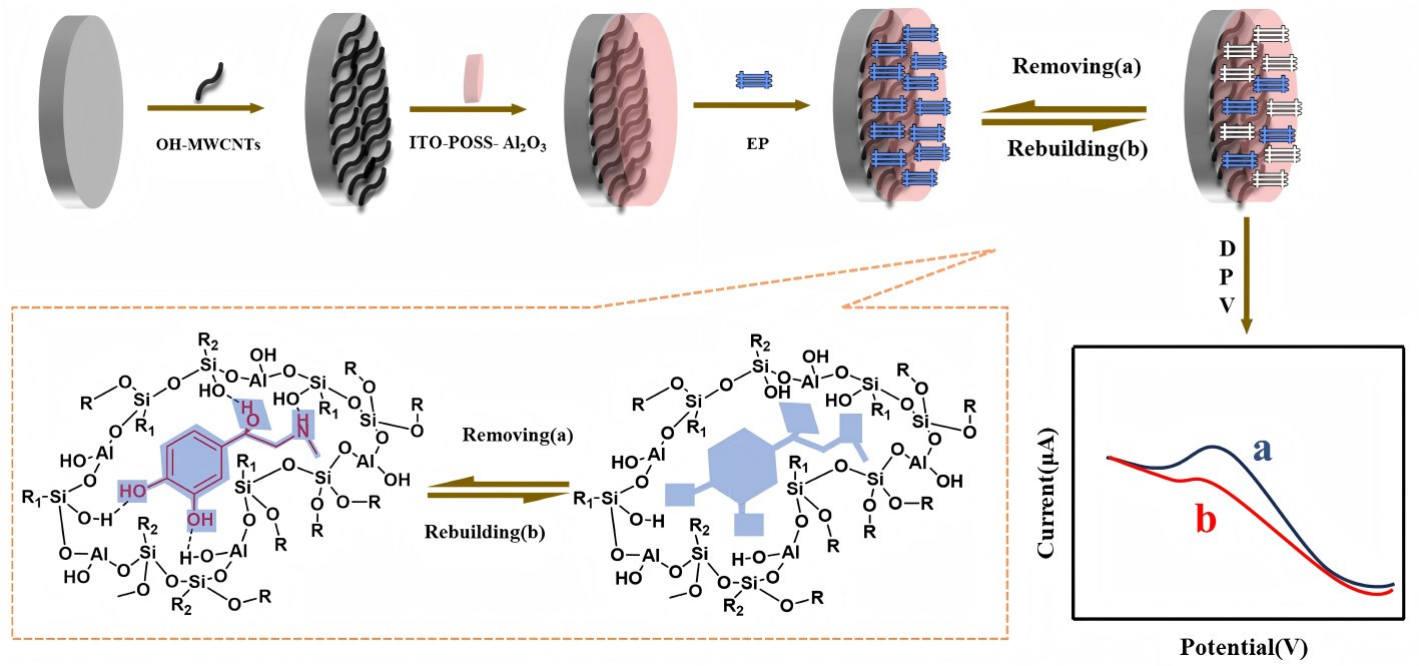
分子印迹电化学传感器检测肾上腺素示意图^［113］^

## 2 绿色分子印迹聚合物的应用

### 2.1 在环境监测中的应用

有毒、有害化学物质的不合理排放加剧了环境污染，影响了水质、空气质量、土壤质量，进而影响人类健康。如何有效监测这些污染物，尤其是种类繁多、物理化学性质差异大且浓度极低的新兴污染物，成为环境监测领域亟待解决的问题。传统的检测方法往往面临成本高、耗时长的痛点，与此同时新兴的荧光纳米传感器因其高灵敏度和选择性在环境监测领域具有巨大潜力，而绿色碳点和MIPs的结合等为开发新型传感器提供了新的可能性^［114-117］^。

近年来，绿色分子印迹聚合物在环境监测领域展现出突破性应用，其核心创新在于结合可持续合成策略与高特异性识别功能。例如环境中有机磷农药^［118］^、阿莫西林^［119］^、甲基红^［120］^和铜离子^［121］^含量的检测，这些突破性进展推动了环境监测向智能化、便携化和零污染方向演进，但复杂基质干扰和规模化生产仍是未来攻克的重点^［122］^。表3列举了GMIPs应用于环境样品中农药残留、易爆物、有机小分子污染物的监测。

**表3:** MIPs在环境监测中的应用示例

Applicable objects	Outstanding advantages	Ref.
Paclobutrazol	high selectivity， physical strength and high stability， high sensitivity	［^123^］
Ultrasensitive dimethoate	high selectivity， excellent chemical stability， recoverability and low cost	［^124^］
Pesticide residues	selective identification ability， wide application， customization， portable detection	［^125^］
Nitro explosives	easy to synthesize， reusable， selective binding	［^126^］
Large molecule and small molecule pollutants	multifunctional， environmentally friendly	［^127^］

### 2.2 在食品安全分析中的应用

随着人类活动的增加，食品样品中的污染物水平正在以惊人的速度上升^［128，129］^，尤其是不当的农业实践导致的农药和除草剂的过度使用^［130］^，这些化学物质的暴露可能引起人类多种健康问题，因此迫切需要在食品消费前检测这些化学物质的存在。传统的检测方法面临灵敏度低、选择性差、检测成本高、样本前处理复杂等问题^［131］^，而磁性分子印迹聚合物^［132，133］^等新兴分子印迹聚合物因其简便的样品前处理、高吸附容量、适用性广等一系列优势为问题的解决提供了新的思路。

近年来，绿色分子印迹聚合物在食品分析领域通过创新设计与功能集成显著提升了检测效率与安全性，检测对象持续扩展，从有机磷农药（OPs）、拟除虫菊酯类杀虫剂、苯并咪唑类杀菌剂到磺酰脲类除草剂（SUHs）、氨基甲酸酯类杀虫剂以及抗生素残留等^［134-137］^，尽管GMIPs在复杂食品基质抗干扰和规模化生产方面仍需优化，但其低毒性、多功能性、对痕量目标分子的高灵敏度检测、在恶劣的化学和物理条件下表现出的良好稳定性^［138］^，为食品安全监测打造了坚实的护盾。表4列举了部分MIPs在食品安全分析中的具体应用示例。

**表4:** MIPs在食品安全分析中的应用示例

Applicable objects	Outstanding advantages	Ref.
Ciprofloxacin	selectivity， sensitivity， stability， simplicity of preparation and cost-effectiveness	［^139^］
Lambda-cyhalothrin	high degree of selectivity， simple preparation process and high practicability	［^140^］
Hydrocortisone	resistant to high pressure and acid alkali corrosion， with fewer operating steps	［^141^］
Thiamphenicol	no need for expensive antibodies or enzymes， simple and cost-effective detection process	［^142^］
Organophosphorus	lower detection limit and quantitative limit， high selectivity and faster equilibrium adsorption time	［^143^］
Norfloxacin	high selectivity， high adsorption capacity， reusable and high magnetic saturation value	［^144^］
Cephalexin	selective and efficient， easy to separate， suitable for analysis of complex samples	［^145^］

### 2.3 在生物医学领域中的应用

近年来，绿色分子印迹聚合物因其环境友好性和精准识别能力在生物医学领域展现出革新潜力。GMIPs因具有物理和化学稳定性、低成本和可重复使用等优点，可以用于酶模拟^［146］^、抗体模拟^［147］^、手性识别^［148］^、生物标志物识别和纯化、微生物检测、药物分析、药物输送系统、激素解毒等^［149-151］^。实际应用时以传感器形式为主，捕获分析物用于其结构评估，通过修改响应信号来发挥作用，这些传感器可分为光学^［152］^、电化学^［153，154］^、压电^［155］^、磁性^［156］^等诸多种类，这些创新表明GMIPs正在重塑精准医疗范式，其绿色合成策略与生物正交响应机制的融合，为个性化治疗开辟了新维度。

Peng课题组^［157］^成功合成了一种光磁双重响应的分子印迹聚合物（DR-MIPs），该材料对磺胺嘧啶（SMZ）具有高选择性和良好的重复性，且DR-MIPs在交替365 nm和430 nm光照下能够实现SMZ的可控释放和再吸附，为抗生素残留物的高效检测和分离提供了创新的技术途径。

甲氨蝶呤（MTX）是一种广泛用于治疗多种恶性肿瘤的药物^［158］^，但其缺乏对肿瘤细胞的选择性，可能导致正常细胞受损，产生严重的副作用。Zhou及其团队成员^［159］^成功制备了磁性分子印迹聚合物，并建立了基于磁性分子印迹固相萃取（MSPE）的高效液相色谱-紫外检测（HPLC-UV）方法，用于血浆中MTX的快速、选择性提取和检测。

雷公藤红素（Cel）是一种存在于中药雷公藤中的天然活性成分，具有显著的抗炎、抗癌、抗氧化和减肥等药理作用。然而，Cel毒副作用显著，可能导致器官损伤甚至急性肾衰竭，因此对其在中药中的含量进行准确检测至关重要。Li等^［160］^开发了一种基于L-半胱氨酸（L-Cys）修饰的锰掺杂硫化锌量子点（L-Cys@Mn-ZnS QDs）并结合分子印迹技术的荧光探针，用于中药中Cel的选择性检测。该探针具有14.19的高印迹因子，在0.1~3.5 μmol/L范围内表现出良好的线性响应，检出限为35.2 nmol/L。此外，Li等^［161］^还提出了一种基于Cu²⁺介导相互作用制备分子印迹聚合物功能化磁性碳纳米管的方法，用于选择性识别中药中的Cel。所制备的Cel-MIPs@MCNTs具有高吸附容量（13.35 μg/mg）、快速动力学平衡时间（40 s）。黄酮类化合物因其抗氧化、抗炎等药理作用而受到广泛关注，槲皮素作为其中一种典型代表，具有清除自由基、抗病毒、抗肿瘤等多种生物活性。Li等^［162］^使用了槲皮素、黄岑素等多种化合物，以及多壁碳纳米管等材料，制备MIPs，并以杨梅素、木犀草素等作为竞争者设计竞争实验，结果显示，MIPs对槲皮素具有更高的结合能力，其印迹因子为8.44，选择性系数分别为12.27、5.66、12.79和9.23。

绿色分子印迹聚合物在生物医学领域中还有其他广泛应用。比如，检测天然抗癌药物紫杉醇^［163］^、蛋白质合成的关键成分色氨酸^［164］^、抑制或杀灭细菌感染的抗生素（如土霉素）^［165］^、用于治疗多种细菌感染的第四代氟喹诺酮类抗菌剂莫西沙星^［166］^，以及广泛用于治疗人类呼吸道疾病的沙丁胺醇^［167］^等。

### 2.4 在样品固相萃取中的应用

传统的液液萃取（LLE）方法需要大量样品和有毒有机溶剂^［168］^，耗时且成本高昂。尽管固相萃取（SPE）^［169，170］^技术在一定程度上解决了这些问题，但其性能往往受限于基质组成，且在提高萃取量和选择性方面存在局限性^［171］^，基于绿色吸附剂的萃取技术包括各种固相萃取的形式如磁性固相萃取、搅拌棒吸附萃取、固相微萃取（SPME）、分散微固相萃取、填充吸附剂微萃取、薄膜微萃取等。MIPs具有新颖的绿色特性，例如高选择性、可重复使用性和高效性，在分离过程中表现出色。

在分子印迹聚合物固相萃取法（MISPE）中有4个重要的步骤，包括调节、加载、洗涤和洗脱^［172］^。离线模式和在线模式是SPE中最基本和最广泛使用的两种模式，需要对这两种模式中的多个参数进行优化。这些参数包括接触时间、样品强度、样品pH、使用的吸附剂量、样品流量、盐和缓冲溶液的加入以及洗涤溶剂、洗脱溶剂和上样溶剂等^［173］^。MIPs作为绿色吸附剂在样品预处理中得到广泛应用。表5列出MIPs在样品固相萃取中的应用示例。

**表5:** MIPs在样品固相萃取中的应用示例

Applicable objects	Outstanding advantages	Ref.
Amphetamines	excellent performance in selectivity， sensitivity， efficiency， and cost-effectiveness	［^174^］
Griseofulvin	simple process， high selectivity， cost-effectiveness， minimal matrix interference， small sample size and solute dosage	［^175^］
Epoxy triglyceride	convenient， with higher repeatability， efficient separation， and environmental friendliness	［^176^］
Phthalate esters	high selectivity， high capacity， and low detection limit， suitable for sensitive determination of trace target analytes in complex samples	［^177^］
Coumarins	high selectivity and extraction efficiency can achieve effective separation and detection of coumarin	［^178^］
Bisphenol A	effective enrichment and detection of BPA， with a imprinting factor of 6.58	［^179^］
Amphetamine Modafinil	highly selective， high-capacity， and fast kinetic properties effectively extract target compounds from complex matrices	［^180^］

### 2.5 在其他方面的应用

除上述应用外，绿色分子印迹聚合物在其他领域也有出现。例如，在法医毒理学方面，MIPs具有巨大的潜力，特别是在样品制备、预浓缩和分析物检测方面，它们的高稳定性和可重复使用性使其成为处理复杂生物和非生物基质的理想选择。然而，MIPs在法医分析中的应用仍处于早期阶段，需要进一步研究来解决合成复杂性、模板泄漏和非特异性结合等问题^［181］^。

在能源储存方面，通过丙烯酰胺接枝壳聚糖并使用环氧氯丙烷（EPI）作为交联剂，Dipali等^［182］^成功合成了一种对镉具有高选择性和高吸附能力的离子印迹聚合物，为从镍镉电池废料中回收镉提供了新思路。此外，分子印迹聚合物在二氧化碳的吸附和分离中也有应用，这些发现对于推动CCUS（碳捕获、利用和储存）技术的绿色发展具有重要意义，为实现净零排放目标提供了新的视角和具体建议。

## 3 绿色分子印迹聚合物的挑战与展望

### 3.1 挑战

绿色分子印迹聚合物在迈向实际应用中仍面临多重挑战。首先，绿色制备方法推广度、应用度不足，而传统的分子印迹聚合物制备方法仍然使用大量的有机溶剂，这与日益严格的化学品使用和排放法规相冲突。其次，复杂环境中的稳定性问题限制了其应用。再者，降解机制研究滞后，现有研究多聚焦于短期性能，缺乏对绿色分子印迹聚合物长期环境归宿的评估，由于分子印迹聚合物的合成方法通常涉及高量的溶剂和烦琐的工艺步骤，这使得商业化进程亦受限。此外，分子印迹聚合物的形态控制较为困难，传统方法难以兼顾高精度与低成本，而新兴的3D打印辅助制备技术虽能实现复杂结构定制，但设备投入与材料兼容性仍是瓶颈。

### 3.2 展望

随着人们对于环境安全的密切关注，更清洁的合成策略正在被推动制定，特别是应用绿色化学和工程原则特异性结构设计成为突破方向，例如，ScCO_2_作为一种绿色溶剂，已在分子印迹聚合物生产中得到应用，它具有成本低、无毒、非易燃、惰性、无味等优点，并且可以容易地去除，无需额外能量输入，在提高结合位点均一性的同时又避免了有机溶剂的使用。采用可再生资源（如纤维素、甲壳素、多巴胺等）作为功能单体或载体，替代传统有机小分子功能单体。随着人工智能（AI）技术的发展和普及，结合绿色化学原则的应用，AI技术有望在分子印迹聚合物的合成和设计工具的优化，以及生产过程的简化，得到更广泛的应用，未来绿色分子印迹聚合物发展大概率会向跨学科方向继续迈进，从分子设计到工程化应用的全链条优化，才能实现绿色化学与精准技术的协同跃迁。
